# Developing an explainable machine learning and fog computing-based visual rating scale for the prediction of dementia progression

**DOI:** 10.1038/s41598-025-06310-4

**Published:** 2025-07-16

**Authors:** Zainab H. Ali, Esraa Hassan, Shimaa Elgamal, Nora El-Rashidy

**Affiliations:** 1 Department of Embedded Network Systems and Technology, Faculty of Artificial Intelligence, Kafrelsheikh University, El-Geish St, Kafrelsheikh, 33516 Egypt; 2 Department of Electronics and Computer Engineering, School of Engineering and Applied Sciences, Nile University, Giza, Egypt; 3 Department of Machine Learning and Information Retrieval, Faculty of Artificial Intelligence, Kafrelsheikh University, El-Geish St, Kafrelsheikh, 33516 Egypt; 4 Faculty of Medicine , kaferelshikh University , kaferelshikh, Egypt

**Keywords:** Machine learning, Mental test scores, Real-time monitoring system, Internet of things, Fog computing, Cardiology, Health care, Engineering

## Abstract

Recently, dementia research has primarily concentrated on using Magnetic Resonance Imaging (MRI) to develop learning models in processing and analyzing brain data. However, these models often cannot provide early detection of affected brain regions. Alternatively, mental test scores such as the Mini-Mental State Examination (MMSE) and Montreal Cognitive Assessment (MoCA) offer valuable insights into the likelihood of dementia and cognitive impairments. The main objective of this study is to introduce an innovative and dependable context-aware health monitoring system based on fog computing to measure mental impairment in the elderly population. The framework provides screening tests utilizing MMSE and MoCA to achieve accurate and real-time monitoring of cognitive function, allowing for early detection and treatment of mental disorders. To assess the effectiveness of our screening test, we evaluated a dataset comprising 450 subjects with Mild Cognitive Impairment (MCI) from Kaferelshikh University. The aggregated dataset is categorized into three classes: (1) 150 patients with MCI, (2) 150 subjects with subcortical diseases, Parkinson’s Disease (PD), and (3) 150 subjects with cortical diseases, Alzheimer’s Disease (AD). To accurately determine health risks, we employ an ensemble AdaBoost model, providing superior performance in accuracy, precision, recall, F-score, and Area Under the Curve (AUC). To validate the effectiveness of our Machine Learning (ML) model on unseen data, we evaluate an additional 18 subjects using the proposed scoring test, with six subjects from each class. The results indicate that our proposed ML model achieves an impressive accuracy of 0.93, outperforming the MoCA score (0.90) and MMSE score (0.83). Through our research, we demonstrate the potential of our context-aware fog computing approach in significantly enhancing early diagnosis of dementia, leveraging mental test scores as valuable indicators.

## Introduction

### Overview

**Fig. 1:**
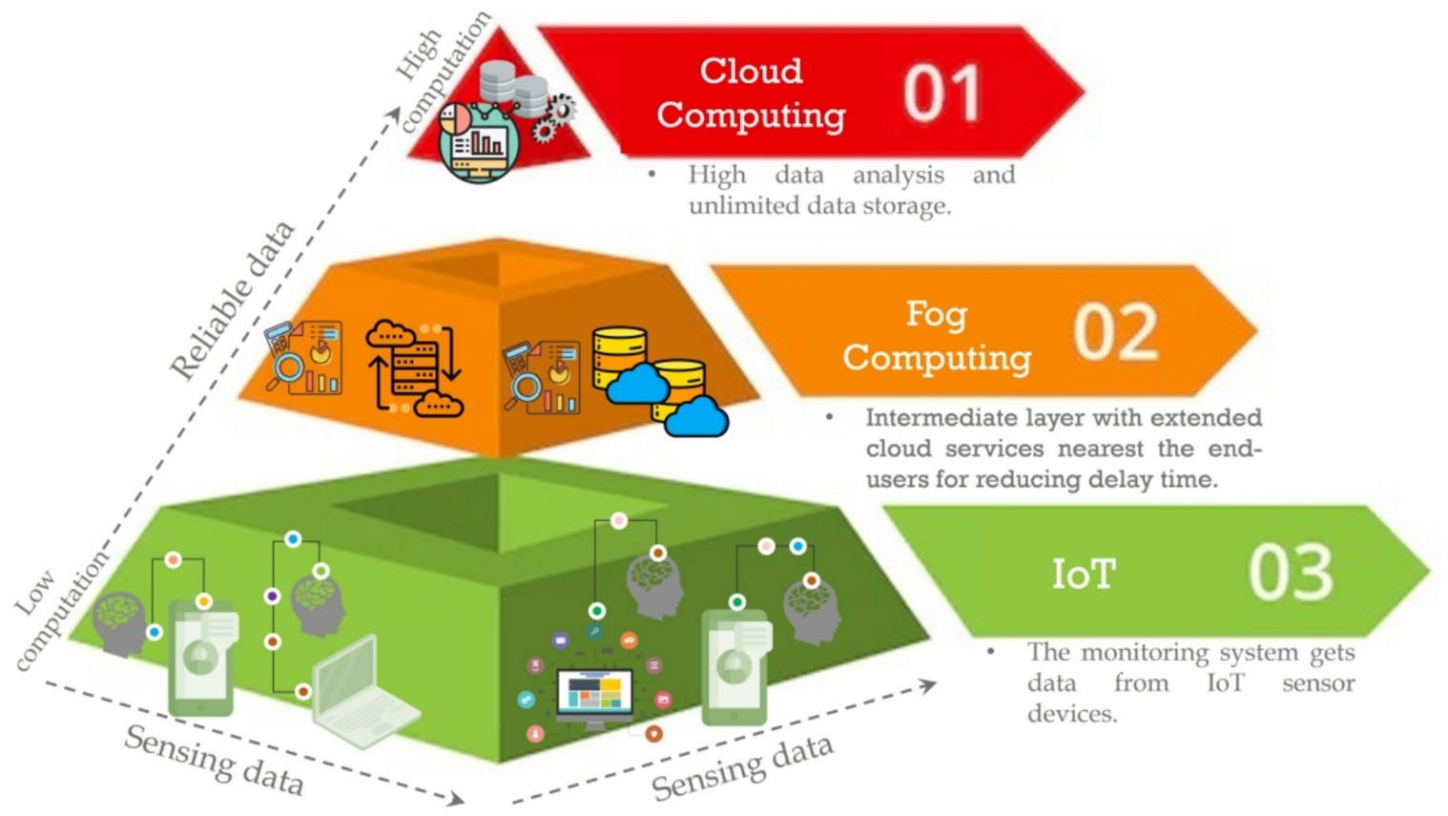
The structure of the hybrid model.

With increased clinician burnout, it is essential to accelerate the growth of telehealth to provide high-quality healthcare services to individuals. Integrating the Internet of Things (IoT) and computing platforms such as fog, edge, and cloud can enhance the quality of healthcare services by enabling various telecom computing solutions. Reliable data collection is one of the biggest challenges in the healthcare industry, and intelligent sensors powered by IoT can help address this challenge seamlessly^1^. With recent advancements in IoT and computing platforms, hybrid modules have been created, delivering efficient e-healthcare computing solutions and enabling easy wireless data transfer among different units. Fog computing is a low-cost computing paradigm that extends cloud services to the network’s edge. Unlike traditional computing services, fog computing provides massive computational and e-services on-demand, with proximity to end-users. Doing so reduces the latency time, and reliable and effective data are handled^2^. Figure 1 illustrates the structure of the hybrid model. Furthermore, a comparison of cloud and fog technologies will be conducted in Table 1.

**Table 1:** A comparison of the features of cloud and fog technologies.

Parameter	Cloud	Fog
Decision-making^2–4^	Remotely	Locally
Data analysis^5,6^	High	Low
Delay time^2,3^	High	Low
Geographical distribution^2,3^	×	√
Transmission reliability^5–7^	√	√
Scalability^5–7^	√	√
Energy consumption^2,4,7^	High	Low
Ubiquitous performance^4,5,7^	√	√
Control and management^4,5,7^	Centralized	Decentralized

Mild Cognitive Impairment (MCI) is a neurocognitive disorder that affects a significant portion of the elderly population. It is characterized by a slight memory deficit, which often describes a transitional state between Cognitively Normal (CN) and overt dementia, such as Alzheimer’s Disease (AD) and Parkinson’s Disease (PD). The symptoms of MCI are not severe enough to interfere with daily activities, but they are noticeable enough to cause concern. Memory loss is a common symptom of MCI, but other cognitive functions, such as decision-making, language, and attention, may also be affected^8,9^.

Based on the clinical trials in the last ten years, cognitive impairment diseases have affected more than 60% of MCI patients; the diagnosis and severity of the disease vary extensively between individuals^10^. Unfortunately, these outcomes’ factors are not fully understood^11^. Medical studies exploring the relationship between cognitive impairment and the risk of developing PD or AD have provided valuable insights into these neurodegenerative diseases. While cognitive impairment is strongly associated with AD, PD also presents cognitive symptoms, albeit with a different cognitive profile. Understanding the nature of cognitive impairment and its relationship to specific neurodegenerative diseases is crucial for accurate diagnosis, appropriate management, and targeted interventions to enhance the quality of life for individuals affected by these conditions^12,13^. Although this impact is significant, it is frequently missing in many studies. Some studies mentioned that mild cognitive impairment mainly depends on memory impairment for predicting AD.

The available information regarding the correlation between cognitive function in domains other than episodic Memory and the hazard of developing AD is limited. While episodic memory deficits are commonly associated with AD, the impact of different cognitive domains on the risk of developing the disease is not yet fully understood^14,15^. Episodic Memory is defined as the ability to remember specific events or experiences, and it is often one of the first cognitive functions affected in AD. However, AD is a complex neurodegenerative disease that involves impairments in multiple cognitive domains, including language, attention, executive function, and visuospatial abilities. Several studies have provided a detailed analysis of the progression from MCI to AD^16–18^, and they conclude reasons as follows: (i) MCI subject has less significant memory defects than AD, (ii) MCI subjects convert to AD within a range of time. Therefore, MCI needs special treatment to stabilize the cognitive function progression, and (iii) the state of AD could be predicted several years before progression.

Recently, there has been extensive clinical research on using statistical techniques in Machine Learning (ML) and Deep Learning (DL) models to provide more efficient analysis and reliable decisions on large datasets. Every day, health centers produce a lot of data. For ML and DL algorithms to produce reliable results that give patients accurate information and quick outcomes regarding their health, a sizable amount of data is required. In healthcare prospects, the importance of ML and DL lies in their ability to handle enormous datasets more effectively without human intervention, and they improve the overall clinician experience at a lower cost.

In the context of early prediction of cognitive decline, ML and DL techniques have been utilized to drive non-traditional and high computational incentive solutions such as magnetic resonance imaging (MRI)^12–17^, biomarkers, and genetic data^19^. For example, authors in^20^ utilized EfficientNet-B2 as a backbone for the building model to predict Alzheimer’s disease- dementia. They first used EfficientNet to extract features from retrained images and then used the DL model to train the classification model. The same is true for^21^, where they utilized a stratified randomization method to predict cognitive decline. In^20^, the authors used a 3d CNN model to make an early diagnosis for Alzheimer’s disease. Although the results seem better, they have often exceeded expectations. Due to the Patient’s measurements in the diagnosis do not always coincide due to several reasons, including (i) the unavailability of all datasets, (ii) the need for continuous aggregating of patient data to track brain image changes which are expensive, and (iii) several signs don’t appear at the early stage of the diseases. To address this issue, we must find alternative ways to provide an accurate and continuous assessment of MCI patients’ cognitive functions.

### Scoring test

Screening and medical examination tools have been developed to follow up on the status of cognitive decline among MCI patients. In 1975, Folstein developed a screening tool in clinical research called the Mini-Mental State Examination (MMSE)^18^. MMSE is the most common examination designed to detect cognitive dysfunction in different contexts, such as Memory, language, and attention. It allows the physician to determine cognitive ability deterioration as a measure of dementia progression and the influence of pharmaceutical therapies. However, due to the large percentage of a calling effect, there is currently debate whether this method can accurately identify cognitive dysfunction in the early stage. In^19^, they investigated MMSE ability as a screening tool for 34 patients with dementia. They concluded that MMSE should be used with extra care due to its mild sensitivity in predicting early deficits of such dementia diseases^3,11^. In 1995, in Montreal, Canada, the researcher developed a new screening tool known as the Montreal Cognitive Assessment (MoCA) to detect cognitive defects at an early stage^22^. MOCA has more sophisticated and broader tasks to check the visuospatial processing, usually in patients with MCI. In^23^, five meta-analyses of Parkinson’s disease patients were examined to investigate how MOCA and MMSE scores are calculated. They concluded that both metrics could be used interchangeably for early diagnosis of Parkinson’s disease. In another study^24^, 149 patients mentioned that MMSE is more sensitive than MOCA as a screening tool for Parkinson’s disease^25^.

1. **MMSE:** MMSE is a screening tool that quickly assesses cognitive performance. It detects cognitive impairment and estimates cognitive evolution over time. MMSE is widely used in epidemiological studies and forms a part of the Dysostotic Interview Schedule (DIS) and epidemiologic catchment Area (ECA). MMSE depends on multiple cognitive domains, including repetition and verbal recall. Attention, calculation language, and visual construction. It consists of various questions with a minimum score of zero and a maximum score of 30 (appendix A shows the content of MMSE questions). These questions, grouped into seven categories, represent different categories. Each category takes a score from 1 to 8. Table 2 shows the distribution of each category with its degree. MMSE was developed to identify people at risk of developing AD, PD, and other forms of dementia. Unless the accepted results were reported using MMSE in some cases, different physicians reported that MMSE is not an accepted tool for MCI evaluation; patients with cognitive impairment perform in the normal range on the MMSE. Therefore, a tool to evaluate MCI. To address this problem, MoCA was developed as a screening tool for patients with mild cognitive complaints.
2. **MoCA:** MoCA is a screening test developed to detect impairment domains commonly occurring in MCI. It is used to evaluate people with symptoms of cognitive decline, such as memory loss, language loss, etc. The initial version of MoCA covered ten domains using an easy and rapid cognitive task. The iterative enhancement of MoCA took place over six years of its usage. The last version of the MoCA test, available at (www.mocatest.org), is a page of 30 points distributed in Table 3. MoCA is a clinical screening tool for Parkinson’s, brain tumors, Alzheimer’s, etc. For comfort reading, the list of frequently used abbreviations is shown in Table 4.

**Table 2:** MMSE features distribution according to points.

Category	Degree
Orientation to time	5
Orientation to the place	5
Registration of three words	3
Attention and calculation	5
Recall three words	3
Language	8
Visual Construction	1
Total	30

**Table 3:** MoCA features distribution according to points.

Category	Degree
Short memory task	5 Points
Clock-drawing task	3 Points
Cube copy task	1 Point
Verbal abstraction task	1 Point
Serial subtraction task	3 Points
Familiarity with two animals	3 Points
Repetition of two complex sentences	2 Points
Familiarity with the animal’s task	3 Points
Confrontation naming task	3 Points
Orientation to time and place	6 points

**Table 4:** Abbreviations list.

Abbreviation	Description
AdaBoost	Adaptive Boosting
AD	Alzheimer’s Disease
CNN	Convolutional Neural Network
ML	Machine Learning
DL	Deep Learning
FS	Feature selection
IoT	Internet of Things
DDC	Dataset Description and Classification
DMMLP	Decision-Making Based on Multilayer Perceptron
E2EFMD	End-to-End Fog-Making A Decision
SVM	Support Vector Machine
SHAP	Shapely Additive Explanations
DT	Decision Tree
MCI	Mild Cognitive Impairment
ANDI	Alzheimer’s Disease Neuroimaging Initiative
OASIS	Open Access Series of Imaging Studies
CNN	Convolutional Neural Network
MRI	Magnetic Resonance Imaging
MMSE	Mini-Mental State Exam
MoCA	Montreal Cognitive Assessment

### Paper contribution

This study introduces a reliable context-aware health monitoring framework based on fog computing to assess mental impairment in older people. This proposed framework provides a remote diagnosis of dementia diseases using fog computing technology and IoT devices. Fog computing receives sensing data from mobile applications or websites and generates real-time warning alerts based on adequate data analysis. The data analysis operation uses a new screening test that combines the strength of MMSE and MOCA to detect any cognitive impairment progression for MCI patients and predict the development of such dementia in the elderly stage. The proposed framework utilizes Adaptive Boosting (AdaBoost) and a multilayer perceptron model (MLP) for predictive operations. AdaBoost is an ML algorithm that boosts the performance of weak learners by sequentially training them and focusing on the misclassified instances to improve overall prediction accuracy. This study employs the AdaBoost MLP model to enhance the framework’s prediction power. The model can make more accurate predictions by leveraging the strengths of multiple weak learners and compensating for their weaknesses. This approach is particularly useful when a single predictive model may not be sufficient to achieve desired results. The following points summarise the paper’s contributions:

1. Introducing a novel context-aware health monitoring framework utilizing fog computing to assess mental impairment in the elderly, combining advanced technologies for accurate real-time cognitive function monitoring. This framework’s real-time tracking and analysis, facilitated by fog computing and IoT sensors, enable early detection of mental impairments, leading to timely interventions and enhanced outcomes.
2. Aggregating dataset for cortical and subcortical diseases is as follows: (1) 150 patients with MCI, (2) 150 subjects with PD (cortical diseases), and (3) 150 subjects with AD (subcortical diseases).
3. Developing a novel screening test using the MMSE and the MoCA for the early prediction of cortical and subcortical diseases in the Patient’s brain.
4. Building an ensemble of multilayer perceptron (MLP) models for classification. Each neural network weak learners utilize includes adaptive learning with gradient descent, adaptive learning with momentum, and conjugate gradient descent. It helps to reduce errors by using several weak classifiers, leading to improvement in speed and performance.
5. Enhanced prediction power by using the AdaBoost MLP model, the framework achieves improved prediction accuracy compared to using a single weak learner. The sequential training process of AdaBoost allows for the combination of multiple weak learners, resulting in a more robust predictive model.
6. Ensuring the effectiveness of the proposed ML model by testing the performance on unseen data. The test data were also aggregated from Kaferelshikh University, 18 subjects (6 subjects for each class).

### Paper organization

The remainder of this article is organized as follows: Section "Discussion" discusses related work and a recent literature review. Section "Literature review" introduces the proposed reliable context-aware health monitoring framework and its functionalities, and describes the used dataset and its characteristics. Section 4 analyzes numerical results; Sect. 5 details system strength limitations. Section "Proposed reliable context-aware health monitoring framework based on fog-computing" provides the paper’s conclusion.

## Literature review

This section presents a thorough review of the literature pertinent to our research topic. By delving into prior studies, theoretical frameworks, and empirical findings, we aim to provide a solid foundation for our research and highlight our study’s significance in advancing the field’s current understanding. The literature review section is divided into three subsections. The first provides a comprehensive overview of existing research and scholarly works relevant to AD and the usage of learning techniques under investigation. The second subsection aims to establish the current state of knowledge in fog computing and cloud services. The third section illustrates the potential benefit of fog technology in healthcare applications.

### Alzheimer’s disease with learning techniques

As age-associated epidemics, AD and PD have garnered significant attention due to their impact on cognitive function and overall brain health. The prevalence of AD increases exponentially with age, ranging from 1% among people between 60 and 64 years to 38% among individuals over 85 years^26^. Similarly, PD is more prevalent in older individuals. Consequently, there is a growing need to understand the progression of these diseases and develop early identification methods^27,28^.

Patients with MCI exhibit memory impairment that exceeds what would be expected for their age, but they do not meet the criteria for a diagnosis of AD^28^. However, individuals with MCI are at a higher risk of developing AD and PD, making early detection crucial^29,30^. Research indicates that the conversion rate from MCI to AD is approximately 15% per year, while the conversion rate from MCI to PD is approximately 12.8%^29,30^. To address the need for early identification and prediction of disease progression, recent research has focused on predicting cognitive function decline and the risk of developing AD and PD. By combining these predictive models, researchers aim to provide valuable insights into disease trajectories and enable timely interventions.

Advancements in data analysis and ML techniques have facilitated the development of predictive models for cognitive decline and disease progression. These models utilize various data sources, including medical records, cognitive assessments, genetic information, and biomarkers, to identify patterns and indicators of disease progression. Recent studies have shown promising results in predicting the progression of cognitive function. Researchers have identified specific biomarkers, genetic factors, and cognitive assessments that can effectively predict the likelihood of transitioning from MCI to AD or PD by analyzing longitudinal data. These predictive models provide valuable information for clinicians and researchers to intervene early and tailor treatment plans accordingly.

Several studies utilized brain images of MCI patients to predict the progression of brain functions and the development of dementia. For example ^31^, utilized the CNN model to explore MRI images and extract the most significant features. These features detect both an individual’s cognitive symptoms and the neurodegeneration process. The classification model is used to analyze the distribution of the extracted features and the influence of each feature on AD diagnosis. They achieved a performance of 80% in terms of classification accuracy. Others in^32^ claimed that MCI is not the reason for AD conversion. They proposed a dual learning and ad-hoc layer model to predict MCI subjects developing AD. This model takes various inputs, including MRI, demographic, genetic, and neurophysiological data.

The model works to two tasks: (i) predict MCI subjects likely to convert to AD and detect AD subjects themselves. They concluded that MRI is the most accurate prediction task technique. Another model proposed a model for predicting MCI progression to AD based on hippocampal MRI features^16^. The proposed model detects the different patterns, then adopts the Classifier in a way that cuts off at a certain threshold of points. In^11^, an accurate DL model was proposed to assess dementia service that applied to functional MRI automatically. They proposed another predictive DL to identify subjects with dementia based on electronic health record (EHR) datasets (i.e., MRI, medical data, lab test data, pharmacy, etc.). The authors claimed that the model helps predict the risk of AD 4–9 years before the onset of the disease.

Several studies have utilized the Alzheimer’s Disease Neuroimaging Initiative (ANDI) data to predict the diagnosis of AD. These studies leverage ML techniques to analyze the demographic and neurophysiological data extracted from ANDI, providing insights into cognitive function and disease differentiation.

In one study conducted by Williams et al.^33^, four ML models, namely Support Vector Machine (SVM), Decision Tree (DT), Neural Network (NN), and Naive Bayes, were employed to predict cognitive functions based on ANDI data. The study concluded that Naive Bayes achieved the highest accuracy in predicting cognitive function. In another study^34^, a deep neural network (DNN) model was designed to differentiate between AD, MCI, and CN subjects using ANDI data. The proposed model achieved validation accuracies of 76%, 85%, and 72% for CN vs. MCI, AD vs. CN, and AD vs. MCI, respectively. In a study by^35^, a recurrent neural network (RNN) with a Long short-term memory (LSTM) autoencoder model was developed to learn and represent cognitive features using ANDI data. The model achieved an accuracy of 85% in evaluating cognitive performance. Similarly, another study^36^ used ML models and autoencoders to predict the conversion from MCI to AD. The proposed model achieved an accuracy of 83.7% in predicting this conversion.

These studies demonstrate the effectiveness of ML techniques and DL models in analyzing ANDI data for predicting AD diagnosis, cognitive function, and disease progression. The results highlight the potential of these models in aiding early identification and personalized interventions for individuals at risk of developing AD or progressing from MCI to AD. However, it is significant to note that while these studies have achieved promising results, further research and validation are necessary to enhance the robustness and generalizability of these predictive models. Large-scale studies involving diverse populations and longitudinal data would contribute to the advancement and clinical implementation of these prediction models based on ANDI data.

Other studies utilized genetic data for prediction. In^37^, they proposed a DL model that worked on features extracted from genetic data to predict the conversion between MCI and AD two years before the onset of AD. This model proves its efficiency in distinguishing between progressive MCI and stable MCI. The same is true in^38,39^. Other studies mentioned that the progression from MCI to AD is associated with different characteristics. For example, in^35^ karlekar et al. depend on changes in language as an initial sign for AD prediction. They utilized Natural Language Processing (NLP) to classify the differences in linguistics from the Deminta bank dataset. They combined three NN models (CNN-LSTM, RNN-LSTM, and CNN). The analysis is based on the derivative saliency technique that rediscovered the language patterns of AD survivors. The proposed model obtained an accuracy of 95.6% and a sensitivity of 97.04%. They concluded that risk factors could include obesity, depression, smoking, hearing loss, and social isolation.

They regarded PD prediction. Several studies used the Patient’s voice dataset to predict the diagnosis of Parkinson’s. For instance, in^25^, the UCI dataset (the Patient’s voice dataset) was used to indicate the diagnosis of PD based on several ML models. They mentioned that SVM with a polynomial kernel gives the best performance in prediction (accuracy = 86% and sensitivity = 88%). In^40^, a random forest algorithm over the dataset, including attributes, is used. They first used PCA to reduce the data dimension; then, the dataset applied random forest to give the best performance (approximately 90%).

Unless the existence of several studies is concerned with the development of such mental cognitive impairments, such as PD and AD, the accuracy of early diagnosis is still limited. This return to several reasons, including (i) structural neuroimages such as MRI and CCT do not provide all the characteristic features that could provide a diagnosis such as neurodegenerative disorder, (ii) functional neuroimages using SPECT may help in the early diagnosis, but it is expensive, (iii) Transcranial Sonography (TCS) has achieved good performance in early diagnosis of Parkinson’s but it not commonly available, (vi) such brain images need to be reported continuously to track the changes in brain characteristics. A summary of the state of the art is detailed in Table 5.

**Table 5:** Summary of the recent research on AD and PD.

Study	Used Dataset	Models	Accuracy	CT & task
Kan et al.^41^	Image dataset	DT, SVM, NN	85%	Binary
Sarxtage^42^	OASIS dataset	KNN, DT	88%	Binary
Shikla^43^	OASIS dataset	DL	92%	Binary
Thailambal^44^	ANDI dataset	NB, LDA	75%	Binary
Ruksh^34^	ANDI dataset	DL M	85%	Multiclass
Spasov^32^	3D image dataset	DL	86%	Binary
Qureshi^37^	DNA dataset	3D-CNNs	92.2%	Binary
Ding^38^	DNA dataset	RNN	89.09%	Binary
Park^39^	DNA dataset	DL	87.5%	Binary

### Fog technology

According to^45^, IoT is a growing market that demands more effective data storage and processing technologies. Fog computing is a solution for managing the expanding number of linked devices. Unlike remote computing, fog computing makes use of local computer resources. It brings the cloud computing paradigm to the network edge, making it ideal for real-time interactions and IoT applications. Fog computing creates a network fabric that connects data sources and storage locations in the cloud or a client’s data center. It is a decentralized computer infrastructure with files, storage, computing, and applications between the data source and the cloud. The requirement for real-time monitoring mainly drives the adoption of fog computing, since it allows for edge analysis close to the data source.

In contrast, cloud computing focuses more on long-term deep analysis because of its sensitivity^46,47^. Fog computing reduces the likelihood of failure by employing various protocols and specifications. Its hierarchical structure provides a more solid framework than the cloud. The goal of fog computing is to relieve the computational burden of cloud computing. Fog computing has become a popular IoT solution because it brings data collection, storage, networking, and analytics closer to the devices and applications that use the network^48^.

In a fog computing system, most computational tasks are performed in a data hub on a smart mobile computer. This approach is particularly advantageous for IoT due to the immense volume of sensor-generated data. Sending all the data produced by a group of sensors to the cloud for processing and analysis is impractical due to the large bandwidth requirements and the slow back-and-forth communication between sensors and the cloud. This delay issue can be critical, especially in applications like vehicle-to-vehicle communication^49^. While cloud computing remains vital for IoT implementations, it is gradually losing its prominence. Fog computing is poised to take over and handle all essential tasks, relegating cloud computing to a secondary role. With the exponential growth of the IoT, a specialized technology is needed to meet its requirements, and fog computing appears to be the most feasible and practical alternative.

### Fog computing in smart healthcare

In healthcare systems, fog computing has emerged as a prominent solution compared to cloud computing. This is primarily due to several advantages it offers, including enhanced security, efficient utilization of network capacity, reduced operational expenses, decreased latency, ease of developing fog applications in mobile environments, and its ability to withstand harsh environmental conditions^50–52^. Cloud computing was utilized to develop a large-scale, compact health-monitoring model to assist a significant number of rural residents in Bangladesh^53^. This scheme enables effective treatment for a diverse population. In^54^, a cloud-based gaming platform was proposed, allowing individuals to perceive emotions through the cloud server, altering the gaming visualization accordingly.

Furthermore ^55^, introduced a cloud-based telemedicine framework that employed various sensors to monitor cardiac function, record electrocardiograms, and measure blood pressure. The collected data was then transferred to the cloud for automated diagnosis and patient notification. In health monitoring, Muhammad et al.^56^ proposed a computerized speech-recognition device that analyzed spectrograms of patient voice signals to detect interlaced derivative sequences, similarly to^57^, which proposed a cloud computing-based system for monitoring PD in Smart Cities, achieving an accuracy rate of 97.2%. This system takes speech signals as input and analyzes them on a cloud server to determine if they originate from a Parkinson’s Patient or a healthy individual. Subsequently, the collected samples are sent to authorized doctors for appropriate medical intervention, and the Patient is informed of the findings and necessary steps to be taken.

## Proposed reliable context-aware health monitoring framework based on fog-computing

**Fig. 2:**
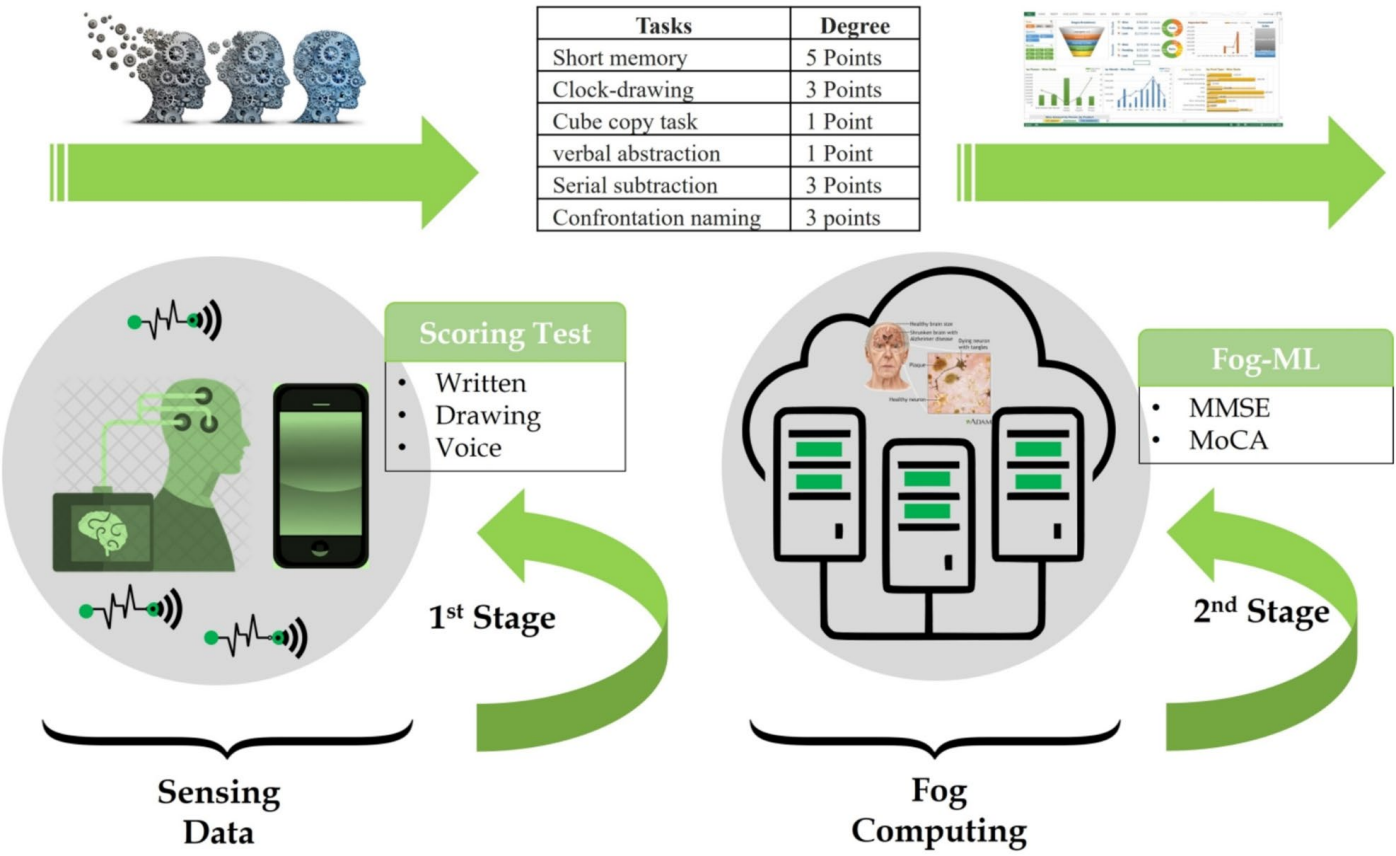
A reliable context-aware health monitoring framework based on fog-computing.

The proposed reliable context-aware health monitoring framework based on fog-computing is specifically tailored to assess mental impairment in elderly individuals. As depicted in Fig. 2, the proposed framework consists of two core stages, including sensing and preprocessing data, and fog-computing, which ensure real-time patient health notifications and alerts through fog-computing and IoT infrastructure. By continuously monitoring patients’ health conditions, collecting data, and performing real-time analysis, the framework aids healthcare providers in identifying patterns associated with dementia diseases. The fog server component provides extensive storage capabilities and generates historical reports while minimizing the transmission of data that requires prolonged analysis and storage. This comprehensive approach contributes to enhanced system reliability and improved network performance.

The framework caters to two types of patients: those already afflicted with the disease and requiring timely monitoring to prevent further deterioration, and those at high risk of developing the disease due to genetic factors or other causes. Data acquisition pertaining to the symptoms of individuals is collected periodically, with predetermined intervals set by healthcare experts. In the cases of both Alzheimer’s and Parkinson’s, patients wear IoT-wearable devices that enable continuous monitoring of their progress and immediate transmission of real-time warnings to healthcare providers.

### Sensing and preprocessing data (SPPD)

SPPD is the initial layer in the first stage, responsible for acquiring real-time data from IoT-wearable devices or personal web communities. The data includes the location sensor, health sensor ID, and environmental information. To avoid heterogeneity and achieve minimal effort and costs of acquired data, the proposed framework adjusts one of the time synchronization cores, such as PTP, PPS, IRIG, and NMEA. At the beginning of the operation, the patients registered themself via the cord number given by the hospital or using Social Security Number (SSN) immediately. The healthcare system can detect the sensor ID by asking the Patient about it; it is unique. Data preprocessing will be locally done at the fog-computing stage to accelerate decision-making and avoid network bandwidth consumption. To put the network bandwidth under control, different communication technologies make a set of communication ranges available for high connectivity among fog nodes (see Table 6).

**Table 6:** The communication ranges.

Technology	Connectivity	Bandwidth	Bit rate	Power consumption	Security
Bluetooth	30 feet	2.4 GHz	280K–420 kbps	1–100	28 bitsAES
Zigbee	10–100 m	868 2.4GHz	20K–250Kbps	–	28bitsAES
RFID	10–20 feet	300 MHz–3 GHz	106K–424Kbps	< 1	High
UWB	Up to25 m	3.1G–10.6GHz	53M–480Mbps	< 1	High
WiFi	50 m	2.4G, G6 GHz	1 M–300 Mbps	> 1000	High

#### Data preparation

The data preparation process in the proposed healthcare monitoring system is divided into three primary operations: (i) outlier detection, (ii) data imputation, and (iii) data scaling. These operations will be discussed in detail in the following subsections.

1. **Outlier Detection**: Outlier detection involves the identification of exceptional values within a dataset in relation to normal values. Recognizing outliers is a crucial step in data preparation as it significantly impacts the performance of classification and clustering algorithms. Various statistical techniques, such as proximity-based models and distance-based methods, address the outlier problem. However, in this investigation, we rely on the expertise of our medical professionals to identify and resolve data outliers.
2. **MICE**: For handling missing values, we employ the Multivariate Imputation by Chained Equations (MICE) method implemented in R. Missing values can arise due to corruption or data collection errors, and their presence can adversely affect classifier performance, leading to bias effects. Basic methods, such as mean, maximum, and minimum, are commonly used for inputting numeric values, while the most frequently occurring item is employed for categorical values. In our study, we observe a small number of missing values in each column, ranging from 2 to 5 for each column. To ensure high accuracy in the imputed data, we adopt the MICE method, which generates n complete datasets by replacing missing variables with n distinct values. These datasets are then analyzed, pooled, and combined to create a resultant dataset. Although this method requires more processing resources, it outperforms single imputation methods^58,59^
3. **Data Scaling**: The purpose of ML data scaling approaches is to normalize all data points. This prevents the Classifier from exhibiting bias towards a particular class, reducing uncertainty and minimizing the likelihood of inaccurate findings or increased cost/processing time. In our study, we employ min–max scaling, which ensures that the data range falls within a specified range. The following equation represents the min–max scaling approach utilized in our research, where $\text{x}$ represents the feature, $\overline{\text{x} }$ represents the feature mean and $\delta$ is the standard deviation^60^
  1 $${\text{X}}^{\prime } = \frac{{{\text{x}} - {\overline{\text{x}}}}}{\delta }$$

The data preparation process in the proposed healthcare monitoring system consists of three primary operations: (i) outlier detection, (ii) data imputation, and (iii) data scaling. These operations will be extensively discussed in the subsequent subsections.

### Dataset description and classification (DDC)

The DDC serves as the primary layer within the fog-computing stage, harnessing the potential of a genuine dataset obtained from real patients. The dataset comprises information encompassing three health-related categories, comprising 150 patients sourced from the neurology clinic at Kafrelskeikh University Hospital. These patients have received clinical diagnoses of AD in accordance with the criteria outlined by the National Institute of Neurological and Communicative Disorders and Stroke-Alzheimer’s Disease and Related Disorders Association (NINCDS-ADRDA)^47^. Several key considerations are essential in this context, including: (i) The patients’ Cognitive deficits and functional impairment needed to be significant enough to satisfy the criteria for dementia. (ii) The definitive diagnosis relied on postmortem examination, ensuring the highest diagnostic accuracy. (iii) For living patients, the clinician’s most reliable diagnosis was classified as probable Alzheimer’s disease. (iv) The clinician diligently ruled out alternative causes of cognitive impairment. These meticulous considerations establish a robust foundation for utilizing the real dataset in further research and analysis within the fog-computing stage, and (v) the cognitive deficits were not operationalized for characteristics or severity.

**Fig. 3:**
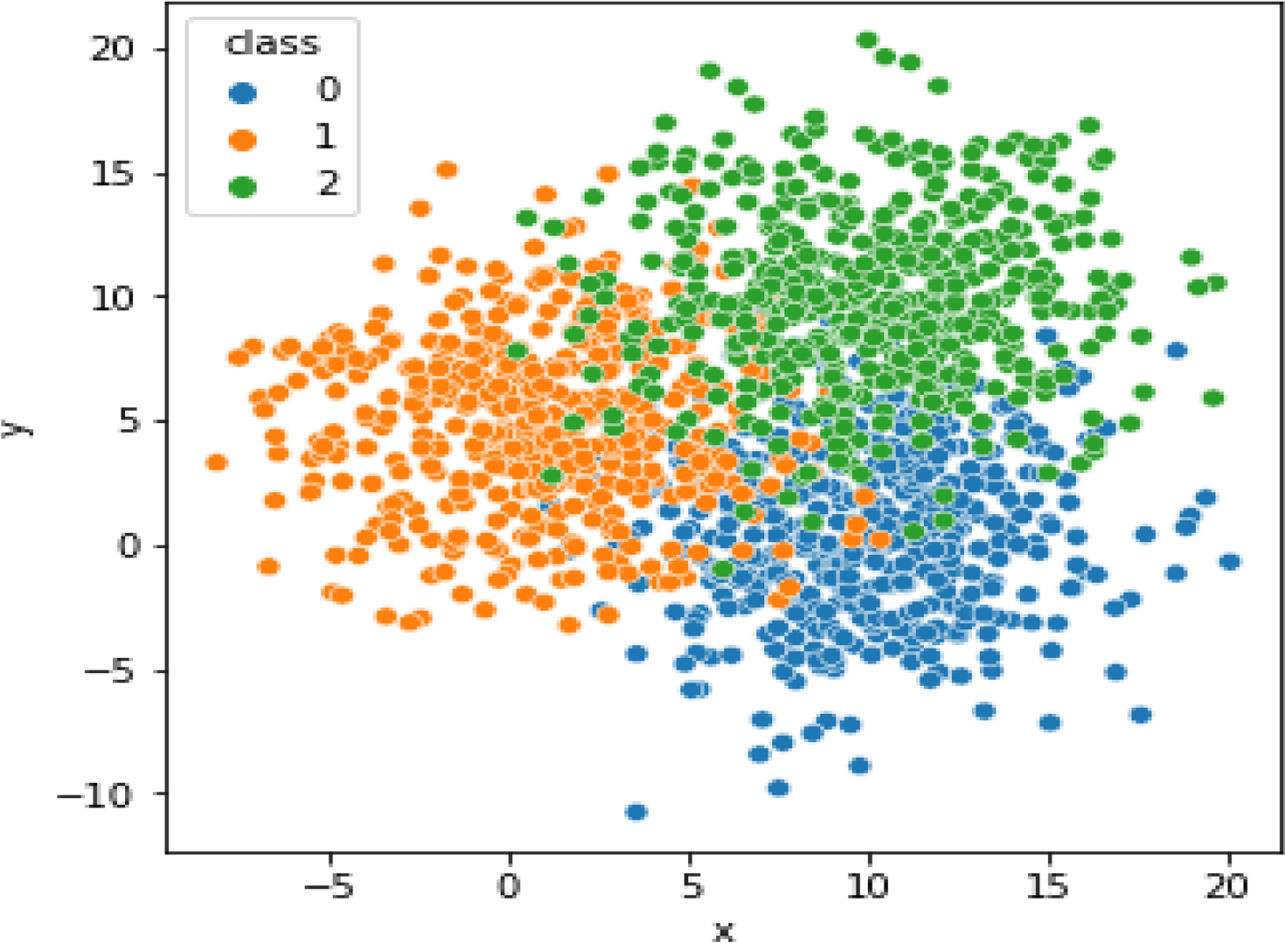
Distribution of dataset. The blue color for Alzahimer class (0), the orang color for Parnksion class (1), and the green color for MCI class (2).

The second category is 150 patients with idiopathic PD with cognitive impairment, admitted to the Neurology department in Kafrelskeikh University Hospital through the outpatient clinic (OPC). All of them were diagnosed according to the UK Parkinson’s Disease Society Brain Bank clinical diagnostic criteria^61^. The third category is 150 patients with MCI (neither demented nor normal). Showed complete general and neurological examination. All patients were examined generally, neurologically, and cognitively by MMNE and MOCA Arabic version to determine the main domains of cognition that are affected in each group for ML for high accuracy of diagnosis and early diagnosis of MCI to manage the case as early as possible and delay dementia whenever possible (see Table 7). Also, all patients underwent routine lab and conventional MRI to exclude any other cause of dementia other than Alzheimer’s or Parkinson’s. The distribution of the dataset is shown in Fig. 3. The distribution of MoCA and MMSE according to parameters is shown in Table 8. The distribution of correct and incorrect samples is detailed in Table 9.

**Table 7:** The fundamental domains of cognition.

Domain Name	MMSE		MoCA	
	Item	Points	Item	Points
Orientation	Orientation to time and place	10	Orientation to time and place	6
Calculation	Subtraction	5	Subtraction	3
Naming	Naming (i.e., pen, glass)		Name (i.e., camel, lion)	3
Repetition	Replete short sentence	1	Replete long sentence	1
Visuoconstructional	Copy pentagon	1	Draw clock	3
			Copy cube	1
Executive function	Reading test	1	Digit span	2
	Step test	3	trial market test	1
Registration	Repeating three words	3	–	
Recall	Recalling three words	3	Recalling four words	4
Writing	Writing one sentence	1	–	–
Attention	–	–	Vigilance test	1

**Table 8:** Distribution of MoCA and MMSE according to parameters.

Context	T MoCA and T MMSE	F MoCA and MMSE	F MoCA and T MMSE	T MoCA and F MMSE	Total	F MoCA Vs. MMSE.p
Attention	131	89	45	185	450	$<0.001$
Naming	196	91	51	112	450	$<0.001$
Calculation	188	78	63	121	450	$<0.001$
Recall	226	62	24	138	450	$<0.001$
Writing	267	88	16	79	450	$<0.001$
Registration	190	95	53	112	450	$<0.001$
Orientation	231	86	20	113	450	$<0.001$
Executive function	237	77	34	102	450	$<0.001$

**Table 9:** Distribution of correct and incorrect samples.

Point of view	Correct MoCA	Incorrect MoCA	Total
Correct MMSE	140	60	200
Incorrect MMSE	200	50	250
Total	240	110	450

### Cognitive progression examination (CPE)

The CPE is the second layer in the fog-computing stage. It provides a new hybrid test based on a set of features extracted from both MMSE and MoCA. The purpose of this hybrid is to provide an efficient monitoring tool for assessing the Patient’s cognitive progression.

The concept of classifying dementia into cortical and subcortical types has garnered considerable attention in both clinical and etiological diagnosis. By studying various neurological conditions that impact specific brain regions, it is possible to identify symptoms such as aphasia, forgetfulness, agnosia, and apraxia as indicative of cortical dementia, which includes AD and frontotemporal dementia. On the other hand, subcortical dementias encompass Parkinson’s disease, Huntington’s disease, Binswanger’s disease, progressive supranuclear palsy, multiple sclerosis, and Sydenham’s chorea. However, certain diseases, such as corticobasal degeneration, multiple system atrophy, vascular dementia, and HIV dementia, affect both cortical and subcortical regions of the brain, resulting in a complex manifestation of dementia^62,63^.

The novelty of the hybrid test is to introduce a set of features combination that allows healthcare providers to evaluate cognitive progression and differentiate between cortical and subcortical diseases, for instance:

1. **Memory test:** since cortical dementia has a more severe loss on the tests of delayed recall, memory testing was chosen (i.e., abnormally rapid forgetting). However, encoding and storage, which are severely compromised in cortical-type diseases, are mostly unaffected. Subcortical abnormalities also result in a deficiency in spontaneous recall.
2. **Language evaluation**: This is attributed to the fact that cortical forms of dementia are more likely to exhibit linguistic impairments. Patients with AD experience progressive language impairments that begin with anomia and advance to transcortical sensory aphasia, eventually leading to a terminal language syndrome characterized by echolalia, logoclonia, and palilalia, resulting in a complex dementia profile^31^. Aphasia manifests early in cortical dementias, such as AD, whereas specific aphasia syndromes have not been delineated in subcortical dementias.
3. Executive functions involve the prefrontal lobes and associated subcortical structures. Therefore, it is reasonable to expect impairments in executive functions in both subcortical and cortical dementias.
4. **Visuospatial function**: Visuospatial deficits are prominent in cortical and subcortical dementias. Although it is challenging to quantify and distinguish cognitive processing slowdown from motor retardation accurately, bradyphrenia appears to be more prevalent in subcortical dementia syndromes than in cortical types^63^. Additionally, 10 tests were employed covering various abilities such as attention, writing, and reading. Table 10 provides an overview of all the score tests, including the hybrid test. These tests were selected under the supervision of a medical expert committee from Kaferelshikh University.

**Table 10:** Features of the new hybrid test.

Feature	Points
Visuospatial and executive	5
Registration	3
Memory	No mark
Naming	3
Attention	6
Language	3
Stage command	3
Reading and obey	1
Writing a complete sentence	1
Delayed recall (recall with cues)	5
Abstraction	2

In AD, the primary pathological features include the presence of senile plaques and neurofibrillary tangles in the cortex, along with generalized cortical atrophy, particularly affecting the frontal and temporal lobes. This degeneration leads to neuronal degeneration and severe brain atrophy. Clinical manifestations commonly observed in AD include dyscalculia, dysphasia, and dyspraxia. On the other hand, subcortical dementia syndromes encompass various conditions such as degenerative extrapyramidal syndromes, subcortical infarctions, acquired immunodeficiency syndrome (AIDS), multiple sclerosis, thalamic degenerative disorders, subcortical trauma, inflammatory processes, and tumors. Lesions in subcortical dementias mainly occur in the basal ganglia, select brainstem nuclei, and the cerebellum^64^. The atrophy of the caudate nucleus, due to its close connections with the prefrontal cortex, can disrupt the caudate-prefrontal loops and lead to neuropsychological alterations in patients without apparent prefrontal lesions. Cognitive impairment in such patients has been strongly associated with the severity of motor symptoms rather than the duration of the disease^65^.

Procedural learning in PD and Huntington’s dementia has been extensively studied. Saint-Cyr et al.^66^ discovered that patients with both conditions exhibited impaired performance on the Tower of Hanoi task. In contrast, amnesic patients with AD typically perform well on this task due to their intact procedural Memory. Executive function, which involves difficulties in verbal fluency, categorization, set-shifting, and planning, is significantly impaired in subcortical dementias^67^. Similarly, it is also affected in early cortical dementia, such as AD. In summary, we aim to develop neuropsychological tests encompassing various cognitive domains to enable clinicians to differentiate between cortical and subcortical dementia even before conducting clinical examinations or investigations.

### Decision-making based on multilayer perceptron (DMMLP)

The DMMLP layer is the third stage of the fog-computing stages, which is responsible for making a real-time informatics decision of health state based on the different attributes of the individual. To this end, we propose an ensemble model based on hybridization between AdaBoost and Multilayer Perceptron (MLP) networks. This proposed model consists of several perceptrons, each a weak classifier with low performance. This results in a more accurate model that outperforms all weak learners. This improvement returns to a couple of reasons, including (i) combining several models results in a more flexible, robust, and precise model, (ii) integration contributes to developing a less biased and more precise model, and (iii) using random subspace and bagging contributes to preventing overfitting.

In DMMLP, the base layer deals with heterogeneous events and the selected proper attributes.

**Fig. 4:**
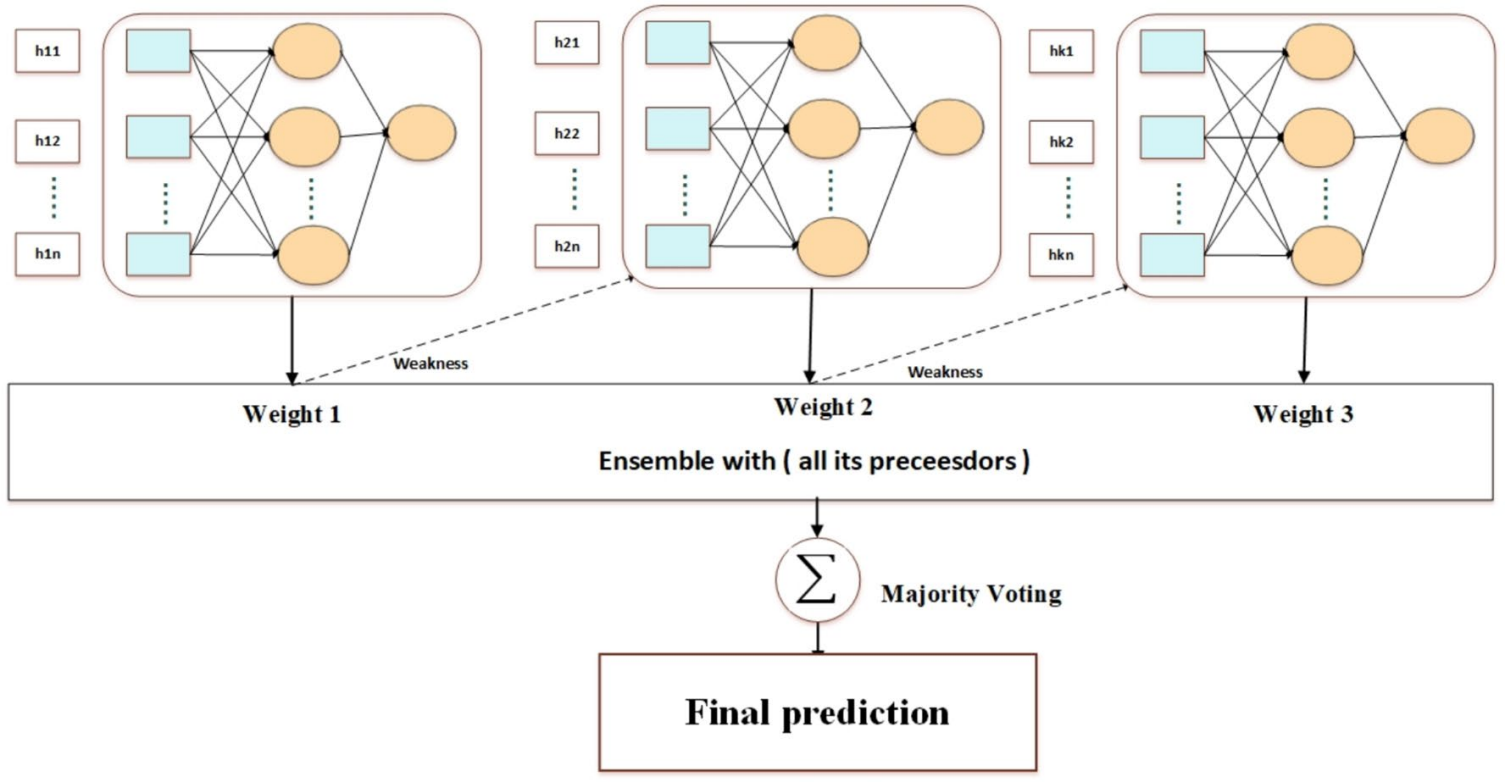
Conceptual of Adaboost MLP model.

1. For each input, the feature vector is mathematically expressed as $X \in {R}^{d}, {y}^{i} \in \{\text{0,1},2\}$, where denotes the vector dimension. {0,1,2} denotes different classes, where d denotes the vector dimension. The classes are denoted as {0, 1, 2}, with 0 representing the Alzheimer class, 1 representing the Parkinson class, and 2 representing the MCI class.
2. Initialize data subsets represented as ${S}_{1 }\left(i\right)=for i=1,\dots ,m$, where $m$ is the number of iterations.
3. As shown in Fig. 4, the predicted states are recognized as ${h}_{t}=pool\{{h}_{1},{h}_{2},{h}_{3},\dots .,{h}_{ns}\}$, where ${h}_{t} \in {R}^{q}$ $q$ denotes the vector’s dimension. The pooling operation combines the outputs of multiple weak classifiers.${h}_{1},{h}_{2},{h}_{3},\dots .,{h}_{ns}$ into a single representation ${h}_{t}$ By applying a specific pooling method (average pooling)^68–70^.
4. The weak classifiers that have the lowest classification error are represented as:
  2 $$C_{{m = E_{{w_{m} }} \left[ {1_{y \ne f\left( x \right)} } \right]}}$$

where $C= 1$ denotes the weak Classifier, and it makes a set of operations as follows:

(1) Training the weak Classifier $C$ on the distribution ${S}_{i}$

(2) Getting the results from the weak Classifier ${h}_{t} :X\to \{\text{0,1},2\}$

(3) choosing the ${h}_{t}$ that result in high performance and low error

(4) $${{\varvec{\varepsilon}}}_{{\varvec{t}}}=prediction \left({S}_{t}\right)\left[{h}_{t}\left({x}_{i}\right)\ne {y}_{i}\right]$$

(5) choosing $$\alpha_{t }$$

3 $$\alpha_{t } = \frac{1}{2}\ln \left( {\frac{{1 - \in_{m} }}{{\epsilon_{m} }}} \right)$$

(6) The weight for all weak classifiers $\theta_{m}$ It is calculated as follows:

4 $$\theta_{m} = \frac{1}{2}\ln \left( {\frac{{1 - \in_{m} }}{{\epsilon_{m} }}} \right)$$

The weight of any classifier with an accuracy of more than 50% is positive, and it becomes higher if the Classifier is more accurate. All classifiers contribute to the final decision. However, the final prediction will not include any classifier with precisely 50% classification accuracy. After each iteration, the misclassified samples are updated with higher weights, and the negatively classified samples behave similarly. All classifiers’ performances are considered when calculating the final decision using the weighted sum process.

### End-to-end fog-making a decision (E2EFMD)

E2EFMD is the final layer at the fog-computing stage; it acts as an IoT presentation layer for replying to individual requests and displaying timely prediction results on smartphones or web applications. This layer also comprises a mapping tool with a Geographical Information System (GIS) to help individuals find the nearest hospital for observation or treatment. Undoubtedly, the advantageous location of fog-computing, which is very close to end-users, reduces the decision delay time and enhances time responsibilities. Algorithm 1 represents the overall operations in the proposed framework.

**Algorithm 1:**
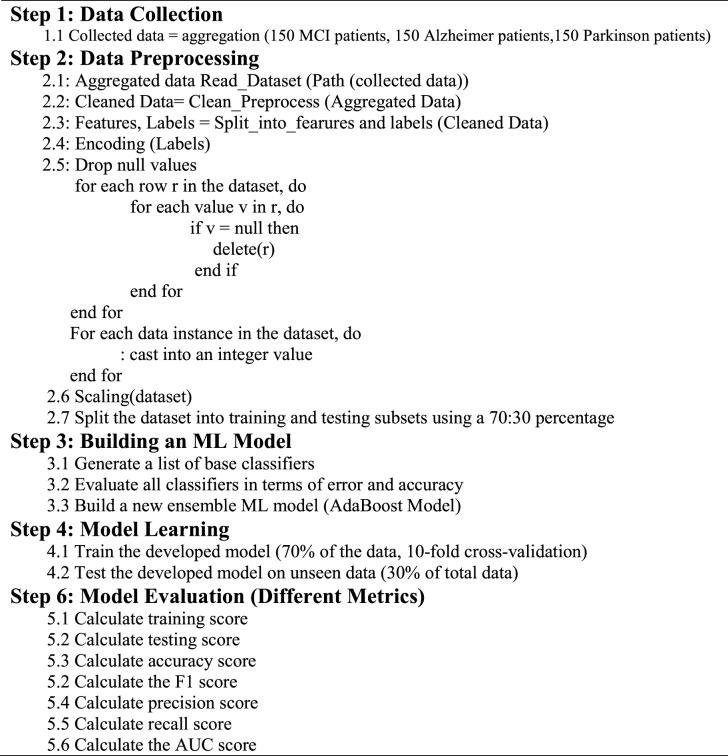
Whole operation of the proposed framework.

## Results and discussion

This section is going to evaluate the proposed reliable context-aware health monitoring framework based on fog computing technology in two directions i) improving network performance in terms of network throughput and transmission reliability and ii) Developing a novel screening test using the MMSE and the MoCA for the early prediction of cortical and subcortical diseases in the patient brain (iii) building new ensemble model that could predict the early diagnosis of Parkinson and Alzheimer diseases based on proposed test results.

### Experiment 1st: comparing fog technology and cloud computing

This subsection contains the experimental results described in the excerpt, which aim to demonstrate the benefits of using fog technology to improve overall network performance. Standard metrics measure network throughput, bandwidth, latency, jitter, and packet loss. The size of the network is between 100 and 500 sensor nodes. The connection between these nodes was made using IEEE 802.11p/WAVE. The configuration of the IEEE 802.11 protocol is described in Table 12, according to the Fog Hierarchical Deployment Model from OpenFog Reference Architecture^65^. The experiment was conducted on an Intel Core i7-1260P, 16 GB of DDR4 storage, 512 GB SSD, and Ubuntu operating system 22.04.1. NS2.35 was used to present and simulate the network model, and the network setting is demonstrated in Table 11. The deployed sensors were reconfigured in a 1:5 ratio^71^.

**Table 11:** Network simulation setting.

Parameter	Value
Network Size	100–500 Sensor
MAC Protocol	IEEE 802.11p
Simulation time	1100 Sec
Antenna Model	OmniAntenna
Channel Type	WirelessPhy
Energy Model	Battery
Packet Size	512 bytes
Traffic Source	CBR

#### Evaluation metrics

**(1) Packet loss:** This metric refers to the number of dropped packets from the source to the destination during data transmission. The percentage of packet loss can be expressed as follows^72^:

$$Packt \,loss\%= \frac{transmitted\, packets\,-\,recieved\, packets}{transmitted\, packets}$$

**Fig. 5:**
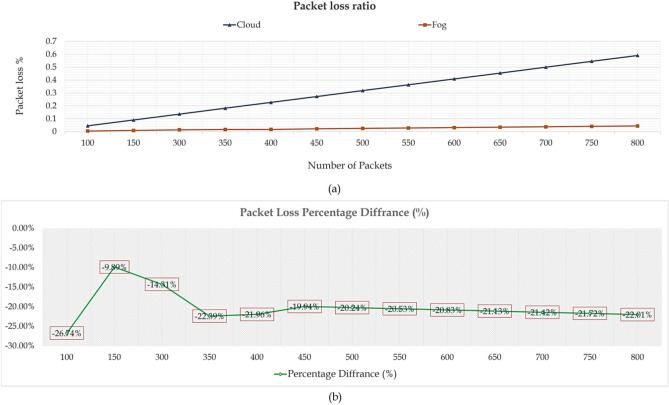
Packet loss percentage vs. the total number of packets.

A comparison of the proposed framework-based fog technology and the existing cloud system^73^ was conducted to test the performance of the proposed architecture in terms of packet loss reduction. As Fig. 5a shows, a significant difference between fog technology and cloud computing is diminishing the number of lost packets across the network. This effect emerged because fog technology’s local data processing feature is crucial in decreasing the need to use network bandwidth continuously. Therefore, there is no network congestion, so the likelihood of losing data and the need to send it frequently is minimal. For more clarification, Fig. 5b shows the difference between fog technology and the existing cloud system (Table 12).

**Table 12:** The configuration of IEEE80211.

Parameter	Value
Setup TxPower	23 bBm/ 18 bBm
Network Bandwidth	20 MHz
Frequency	5.875 GHz
Receiver Sensitivity	− 95.2 bBm
Antenna Gain	2bBi

**(2) Network throughput:** This metric indicates the quality of the network bandwidth or the link quality that is usually used to evaluate the amount of network connection and transmission reliability. The total network throughput refers to the average of successful packet delivery across network channels, and it can be measured as follows^72^:

$$Total \,throughput= \frac{delivered\, packets\,*\,size\, of\, packet}{simulation\, time}$$

**Fig. 6:**
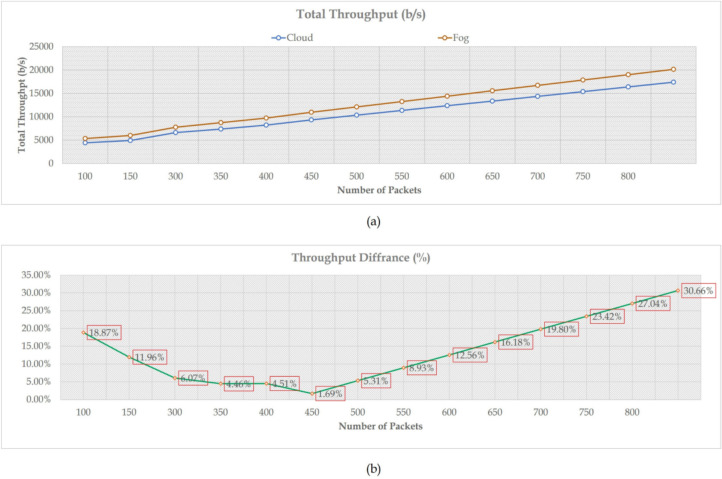
The total network throughput vs. the total number of packets.

Figure 6a shows that the proposed framework-based fog technology was compared with the existing cloud system^73^ to measure its effectiveness in network throughput. The concept of throughput is usually related to both the link quality and the percentage of packet loss. This effect explains that packet loss directly reduces throughput, as some protocols used in the transport layer interpret loss as an indication of link congestion. Thus, their transmission rate must be adjusted to avert congestive network collapse. This describes the proposed framework’s outperformance over the cloud system. The proposed framework maintains its performance by reducing bandwidth consumption due to its ability to deal with packet requests locally without uploading them to the cloud server. For more clarification, Fig. 6b depicts the percentage difference between the two systems.

### Experiment 2nd

#### Experiment setup

All experiments in this study were implemented with an Intel Core i7 workstation with 32 GB RAM and a 2 TB hard disk. We used Python 3.8, which was distributed in Anaconda 3. All developed models were implemented using the Keras library developed on the TensorFlow backend. We used a grid search algorithm to tune all algorithms based on the validation dataset. The best model for each algorithm was chosen based on model performance on the validation data. The final result was calculated based on the test data, which was used sparingly to avoid test set leakage.

#### Evaluation metrics

The effectiveness of the proposed intrusion detection method was assessed using a confusion matrix comprising True Positive (TP), True Negative (TN), False Positive (FP), and False Negative (FN) values.

1. True Positive (TP): Number of records correctly identified as an injection attack.
2. True Negative (TN): Number of records accurately classified as belonging to the regular class.
3. False Positive (FP): Number of records erroneously classified as an injection attack.
4. False Negative (FN): Number of injection attacks that went undetected by the intrusion detection system (IDS).

To evaluate the performance of our proposed model, we employed various metrics, including accuracy, precision, recall, F1 score, and area under the curve (AUC). Cross-validation (CV) results were computed using the training data, while the generalization performance was assessed using the testing data. Detailed information regarding the evaluation metrics used can be found in Table 13.

**Table 13:** Evaluation metrics description.

Metric	Abb	Eq	Description
Accuracy	$ACC$	$\frac{tp+tn}{tp+fp+tn+fn}$	The accuracy is calculated as the proportion of correctly classified cases out of the total cases
Precision	$P$	$\frac{tn}{tn+fp}$	The positive rate represents the percentage of cases of the hostile class that are accurately classified
Recall	$R$	$\frac{tn}{tn+fn}$	The false negative rate is determined by the ratio of correctly classified positive records to the total number of classifications within the class. A higher false negative rate implies a lower detection rate for positive cases
F1- score	F1	$\frac{2(P*R)}{P+R}$	The F1 score, the harmonic mean of precision and recall, is a robust evaluation measure for imbalanced data

### Experiments using MMSE

**Fig. 7:**
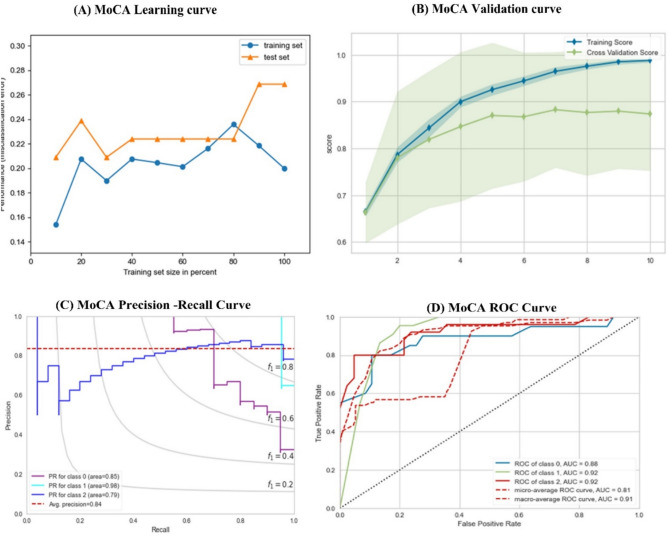
(**A**) MMSE learning curve, (**B**) MMSE validation Curve, (**C**) Percision Recall Curve, (**D**) MMSE ROC Curve.

This section evaluates five single classifiers and the proposed voting classifier. We carefully choose the algorithms that are commonly used on the medical side. As we can observe, DT gives a minor performance with ( ACC = 0.756%, AUC = 0.7571%), followed by KNN with (ACC = 0.822%, AUC = 0.8102%). Unless LR gives the best performance in terms of a training score of 0.8992%, it provides in terms of (ACC = 0.8266%, AUC = 0.8118%). The best performance of the traditional ML models is from MLP (ACC = 0.8531, AUC = 0.8325). Our proposed algorithm outperforms all other algorithms by about 5–7%. It gives the best performance of (ACC = 0.8662, AUC = 0.8425). Table 13 details the results of MMSE. Several figures have been utilized to visualize the proposed model’s performance across the MMSE dataset, as shown in Fig. 6a,b,c,d. Figure 7b shows the relationship between the training data and the cross-validation data as an estimator for the training dataset. The validation curve also helps to identify whether the model is sensitive to bias or variance. However, the learning curve is drawn with the mean score; cross-validation variability is depicted by the shaded areas, representing the standard deviation and mean.

Figure 7b shows the precision-recall curve to evaluate the model’s quality. It represents the tradeoff between the Classifier’s precision (relevance) and recalls (completeness). In our classification task ( multiclass classification task), the curve was created in terms of OneVsRest to produce a binary comparison between classes. The possible instances are all instances related to the class, and the negative is any other instance. Then we used Micro-average to compute the precision-recall curve for all classes. Figure 7D shows the receiver operating characteristic curve; it measures the Classifier’s predictive quality in sensitivity and specificity. The ROC curve displays the TPR on the Y-axis and FPR on the X-axis, resulting in the area under the ROC curve (AUC). AUC is a measurement of the correlation between true and false favorable rates. Table 14 shows the details of the MMSE results.

**Table 14:** Results of MMSE screening test.

Algorithm	Training Score	Testing Score	ACC	Precision	Recall	F-Score	AUC
LR	0.8692	0.8227	0.8266	0.8227	0.7674	0.8133	0.8118
SVC	0.8643	0.8351	0.8352	0.8221	0.8352	0.8311	0.8314
KNN	0,.822	0.8021	0.8366	0.8127	0.7544	0.8257	0.8102
DT	0.7791	0.7590	0.7590	0.7561	0.7192	0.7572	0.7571
MLP	0.8531	0.8521	0.8461	0.8452	0.817	0.8392	0.8325
Proposed method	0.8721	0.8425	0.8662	0.8652	0.827	0.8572	0.8425

### Experiments using MOCA

**Fig. 8:**
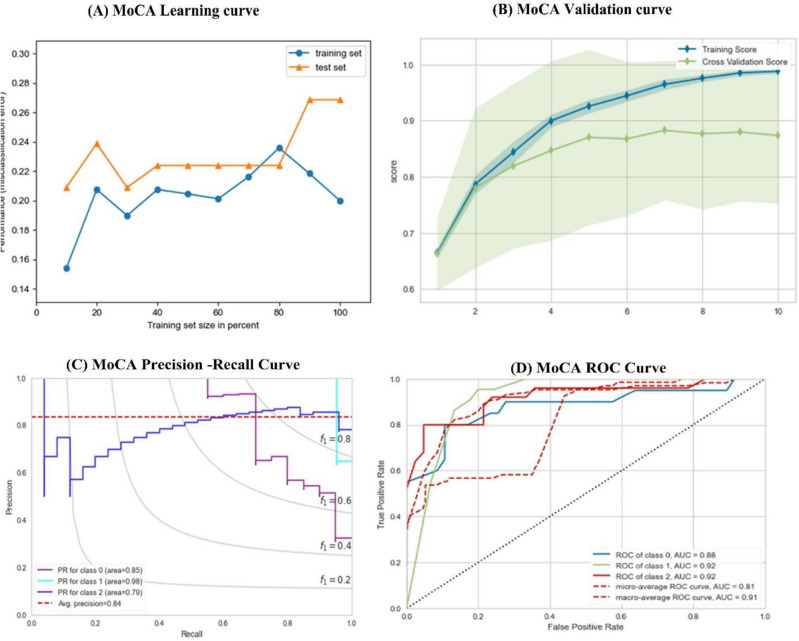
(**A**) MoCA learning curve, (**B**) MoCA validation Curve, (**C**) MoCA Precision-Recall Curve, (**D**) MoCA ROC Curve.

This section evaluates the models based on the MoCA dataset, as seen in Table 14. Using the MoCa dataset increases the performance by about 2% to 8%. SVC gives a minor performance among all traditional classifiers ( ACC = 0.821, AUC = 0.846), followed by DT with the performance of (ACC = 0.832, AUC = 0.885). The best performance from traditional ML was obtained from MLP ( ACC = 0.863, AUC = 0.901) and LR (ACC = 0.8531, AUC = 0.992). The proposed method outperforms all traditional models with differences ranging from 2 to 7%. It achieved 0.901 and 0.915 in terms of ACC and AUC. The proposed method improved when using the MoCA dataset over the MMSE dataset by about 3%. Table 15 shows the results of the MoCA test (Fig. 8).

**Table 15:** Results according to MoCA scoring test.

Algo	Training Score	Testing Score	ACC	Precision	Recall	F-Score	AUC
LR	0.886 ± 0.002	0.855 ± 0.001	0.8531 ± 0.02	0.858 ± 0.012	0.8059 ± 0.001	0.835 ± 0.002	0.882 ± 0.001
SVC	0.854 ± 0.021	0.829 ± 0.023	0.828 ± 0.001	0.833 ± 0.001	0.823 ± 0.002	0.832 ± 0.012	0.846 ± 0.001
KNN	0.854 ± 0.011	0.820 ± 0.001	0.831 ± 0.012	0.830 ± 0.002	0.839 ± 0.021	0.819 ± 0.001	0.866 ± 0.021
DT	0.853 ± 0.011	0.852 ± 0.002	0.834 ± 0.003	0.853 ± 0.001	0.833 ± 0.011	0.843 ± 0.031	0.881 ± 0.011
MLP	0.901 ± 0.013	0.861 ± 0.012	0.842 ± 0.011	0.836 ± 0.021	0.804 ± 0.011	0.836 ± 0.019	0.901 ± 0.035
Proposed Method	0.922 ± 0.0021	0.901 ± 0.001	0.902 ± 0.031	0.911 ± 0.022	0.900 ± 0.039	0.898 ± 0.011	0.915 ± 0.011

### Experiments proposed screening test

This section evaluates the models based on the proposed hybrid score in the aggregated dataset, as we observe in Table 15. The performance increases by about (3% to 9%) over MMSE and (2% to 7%) over MoCA. The minor performance among all traditional classifiers is LR (ACC = 0.805, AUC = 0.852), followed by SVC with a performance of (ACC = 0.871, AUC = 0.906). The best performance from traditional ML was obtained from MLP (ACC = 0.926, AUC = 0.925) and LR (ACC = 0.8531, AUC = 0.992). The proposed method outperforms all traditional models with differences ranging from 4 to 11%. It achieved 0.962 and 0.971 in terms of ACC and AUC. The proposed method improved and outperformed the MoCA MMSE dataset with the same dataset. The performance of the proposed model has been shown in Fig. 7A–D. Table 16 shows the results of the proposed test.

**Table 16:** Results according to the proposed test.

Algo	Training Score	Testing Score	ACC	Precision	Recall	F-Score	AUC
LR	0.853 ± 0.011	0.843 ± 0.001	0.805 ± 0.001	0.858 ± 0.012	0.8059 ± 0.001	0.805 ± 0.002	0.852 ± 0.001
SVC	0.871 ± 0.011	0.862 ± 0.022	0.8524 ± 0.011	0.833 ± 0.001	0.823 ± 0.002	0.833 ± 0.002	0.906 ± 0.001
KNN	0.893 ± 0.012	0.888 ± 0.012	0.866 ± 0.001	0.878 ± 0.001	0.827 ± 0.011	0.856 ± 0.011	0.947 ± 0.031
DT	0.890 ± 0.001	0.882 ± 0.007	0.862 ± 0.015	0.864 ± 0.015	0.848 ± 0.011	0.831 ± 0.001	0.885 ± 0.033
MLP	0.926 ± 0.013	0.911 ± 0.001	0.892 ± 0.002	0.916 ± 0.011	0.882 ± 0.011	0.866 ± 0.012	0.925 ± 0.025
Proposed Method	0.962 ± 0.021	0.932 ± 0.013	0.931 ± 0.001	0.941 ± 0.022	0.900 ± 0.039	0.928 ± 0.011	0.971 ± 0.025

**Fig. 9:**
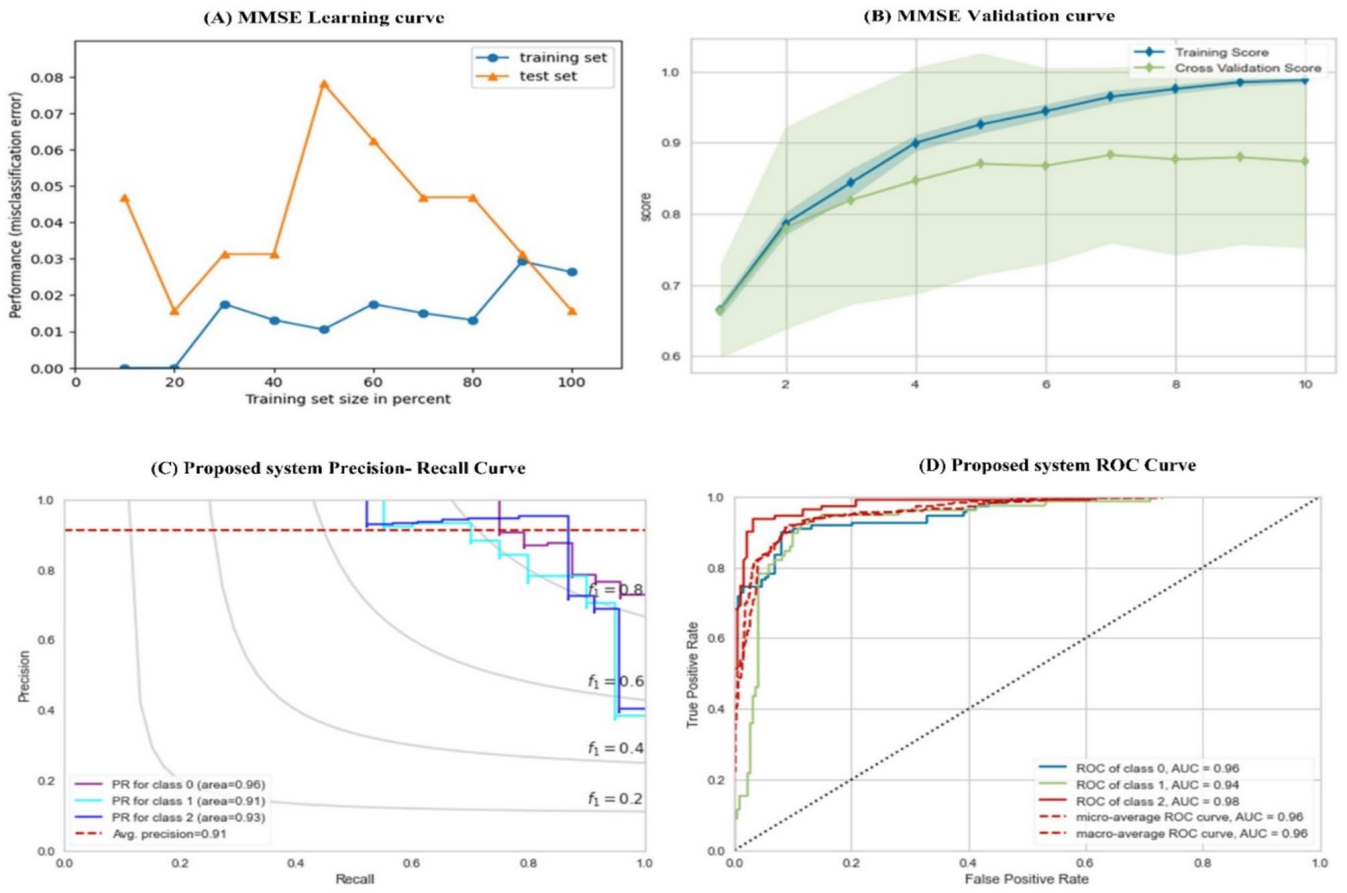
(**A**) Proposed score learning curve, (**B**) Proposed score validation Curve, (**C**) Proposed score Precision-Recall Curve, (**D**) Proposed score ROC Curve.

**Fig. 10:**
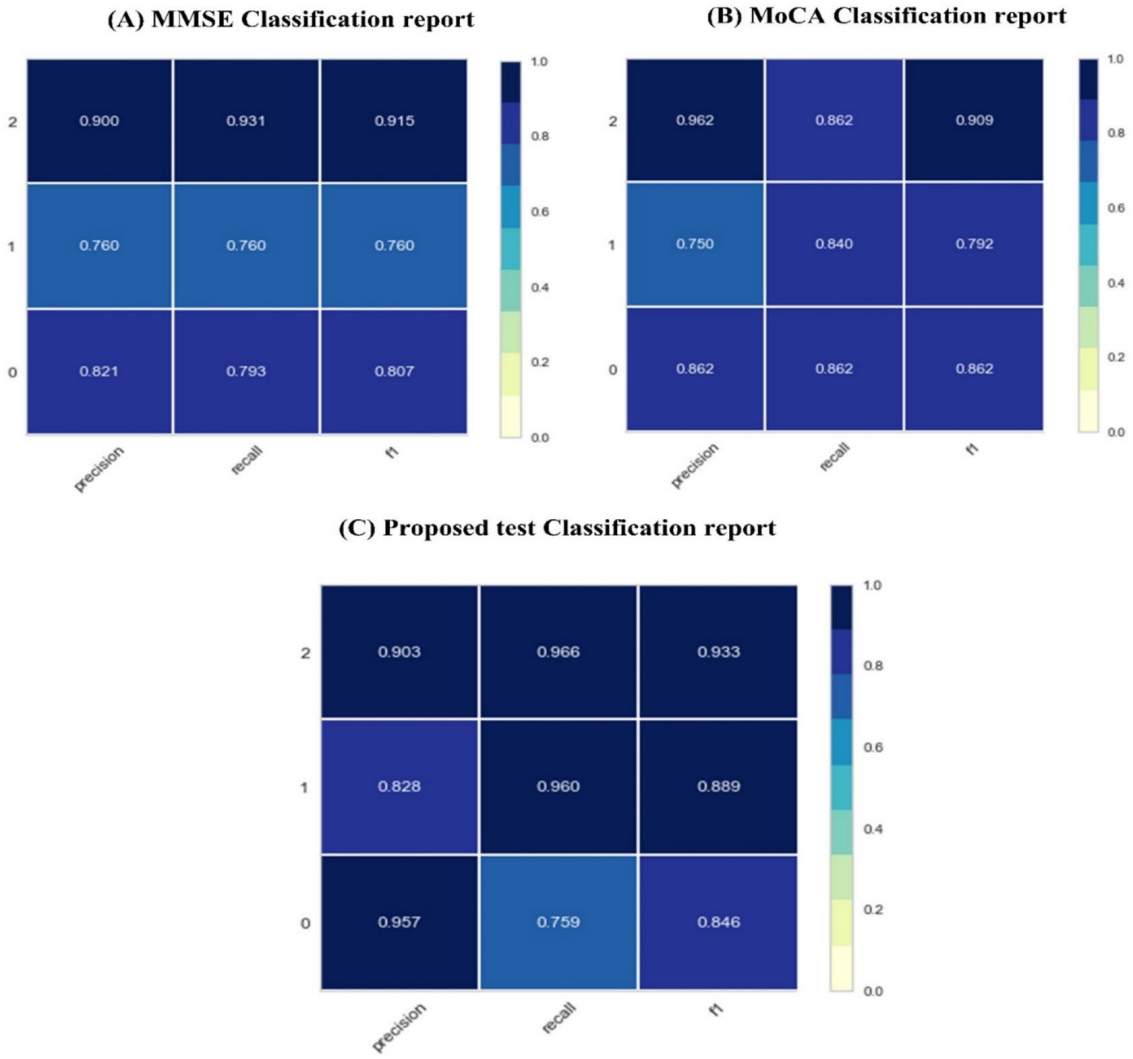
The classification report of scoring tests (**A**) MMSE, (**B**) MoCA, (**C**) Proposed test.

To support a more straightforward interpretation and better comparison across all models, the classification report will describe the numerical results with a colored heatmap. As shown in Fig. 9, all heatmaps ranged from (0.0 to 1.0). These metrics are defined as false negatives, true negatives, false positives, and true positives to show the main classification result per class. By contrast, the model accuracy can hide the functional deficiencies in one class of multiclass problems, and this report provides deeper insights into the model’s behavior. From Fig. 9A, we can observe the following with the classification report of MMSE. (1) class 0 provides precision = 0.821 and recall = 0.793, which indicates that the model of all instances that are classified as PD is correct, where not all PD instances are classified correctly, for class 1(AD class), both negative and positive instances are classified with close percentage (precision = 0.760), and (recall = 0.761), where MMSE is successful in MCI classification (Class 2) (precision = 0.900, recall = 0.931). Figure 9B is the classification report per class for the MoCA dataset. We can observe that the MoCA is less sensitive in classifying PD and AD. MoCA achieved adequate performance in detecting PD (precision = 0.862, recall = 0.862) and AD (precision = 0.750, recall = 0.840). The proposed framework outperforms both MoCA and MMSE tests in all classes. It achieved precision = 0.957, recall = 0.759 in class 0, precision 0.828, and recall 0.960 in class 1. From these results, we can observe the following: (i) MMSE are not sufficient for predicting PD, (ii) MoCA outperforms MMSE in predicting both PD and AD, (iii) MMSE and MoCA are approximately the same in predicting MCI, and (iv) proposed test outperforms both MMSE and MoCA tests in classifying PD and AD. Figure 10 compares the three cognitive tests regarding various evaluation metrics, including precision, recall, measure, etc..

### Statistical significance test

To ensure the superiority of the proposed model, we decided to depend on both accurate results and statistical tests. First, we rely on the Wilcoxon signed-rank test^74^. It’s a nonparametric test developed by Demsar to compare the performance of all algorithms. It counts the number of ties and wins obtained from the algorithm. The algorithm is considered better from a statistical point of view if the number of wins is greater than the number of ties. All algorithms are then compared according to the Friedman test^75^. The Friedman test is a nonparametric test that measures ANOVA. The Friedman test determined whether there was a significant difference between all utilized algorithms, but it didn’t specify the best algorithms. To rank all utilized algorithms and choose the best one, we decided on the Nemenyi test^76^ and calculated the average rank for each Classifier. When several models are compared against each other, the results could be plotted using a critical distance diagram. Figure 10 shows the essential distance of all classifiers according to the average rank of the Nemenyi test.

### Explanation of the proposed hybrid test

**Fig. 11:**
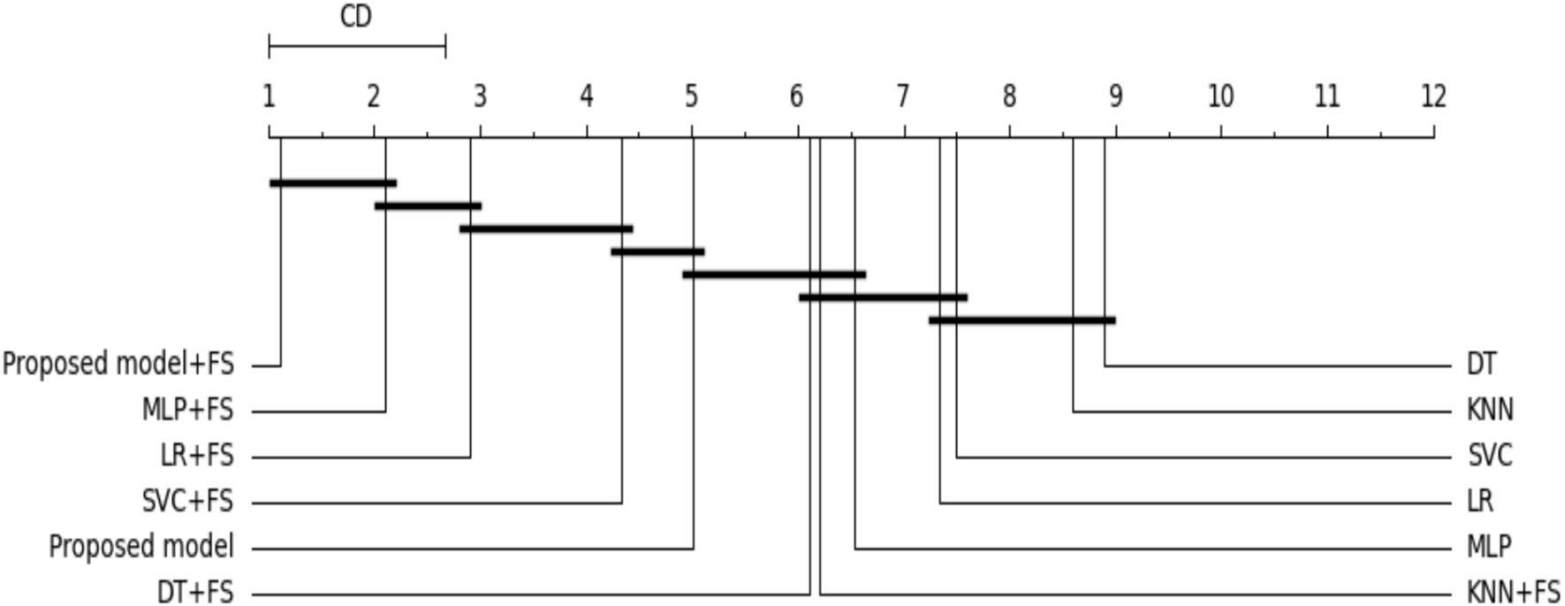
Critical distance between all algorithms.

**Fig. 12:**
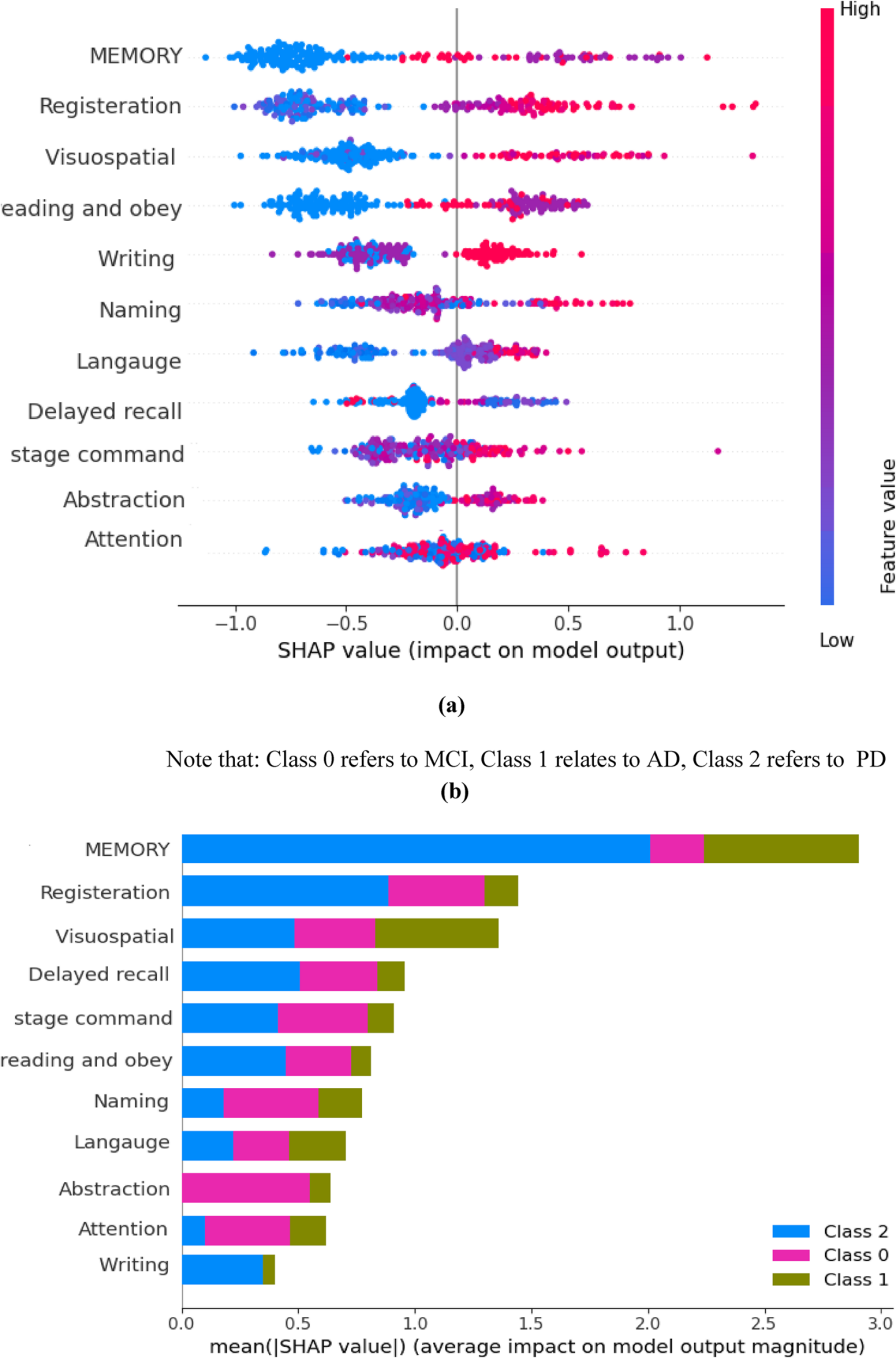
A global explanation using SHAP (**a**) SHAP summary plot using SHAP values (**b**) SHAP plot average impact of all features in each class .

Explanatory tools are utilized to interpret any ML or DL model. It could be done based on two levels: global and local. The global explanation refers to how the full features affect the overall model. In contrast, the local explanation shows that the features with specific values in each instance affect the overall output. SHAP( Shapely Additive Explanations) is one of the valuable tools that could provide each feature’s importance according to the SHAP score^64^. First, we generate a summary plot (Fig. 11a) that visualizes each feature’s contribution to the model’s overall decision. The plot displays a bar graph with the critical features on the x-axis and their corresponding feature importance on the y-axis. The length of each bar represents its importance. The blue indicates the model’s contribution to moving the model toward a positive class, while the red indicates its contribution toward negative courses. Figure 11a shows that naming, registration, and visuospatial are the most impactful features. This plot helps identify the model’s most critical features and how they contribute to the classification of the input data. These results are assured using Fig. 11b. This figure shows how each model affected the class. On the x-axis of the plot, we have the names of the features, while the bars on the y-axis represent the importance of each feature. The color scheme uses blue, green, and purple to indicate the impact of the features on the PD, MCI, and AD classes, respectively. Figure 12a shows that Memory and registration significantly impact the prediction of the PD class but are less critical for the MCI and AD classes. On the other hand, abstraction and attention are highly impactful in predicting the MCI class but have less importance for the PD class and no impact on the AD class.

**Fig. 13:**
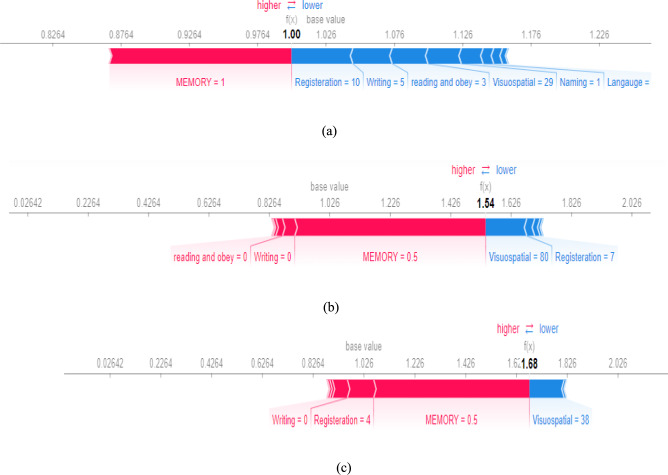
A global explanation using SHAP (**a**) SHAP summary plot using SHAP values (**b**) SHAP plot average impact of all features in each class .

To examine the significance of each feature for individual instances, we used SHAP local explainers for local explanations[77]. Figure 12a shows that the base value was 1.026, and the predicted probability was 1. According to SHAP, Memory, registration, and writing were the most significant features, consistent with the global explainer in Fig. 12. For Fig. 13a–c, the base value was 1.026, and the predicted probability was 1.54. The base value shows the probability that it will go out if this instance has no specific value. Red and blue features show which features contribute to the model in the lower and higher directions.

By combining global and local explanations, we gain a comprehensive understanding of model behavior. Globally, we identify which features are most influential across all predictions. Locally, we dissect individual cases to see how specific feature values alter outcomes. his dual approach ensures model transparency, helps in feature selection, and builds trust in AI-driven decisions, particularly in critical domains like healthcare (e.g., predicting neurodegenerative diseases).

### Case study

In this section, the experiment was conducted on 30 patients at Kafrelsheikh Hospital University to evaluate the performance of the proposed screening test. The steps for assessing the proposed screening test can be summarized as follows: (i) all patients were examined generally to follow up on their cognitive abilities, (ii) patients underwent routine lab and conventional MRI to exclude any other cause of dementia other than Alzheimer’s or Parkinson’s diseases, and (iii)to compare the performance of the proposed screening test with the MoCA and MMSE examination test, all patients were examined with MMNE, MOCA Arabic version, in addition to our proposed test. Results of all examination tests were recorded to provide a fair comparison according to the Patient’s score. Table 17 shows the patient distribution according to their scores in each examination test. Tables 18 and 19 compare the proposed test, MMSE, and MoCA regarding corrected and uncorrected classified samples.

**Table 17:** Distribution of the number of patients according to different scores.

Degrees	MMSE	MoCA	Proposed test (hybrid test)
< 10	1	3	6
≥ 10 and < 15	2	5	7
≥ 15 and < 20	4	7	9
≥ 20 and < 25	4	8	10
≥ 25 and < 30	4	8	13

**Table 18:** A comparison between MMSE versus proposed test.

	Correct proposed test	Incorrect proposed test	Total
Correct MMSE	10	2	12
Incorrect MMSE	16	2	18
Total	26	4	30

**Table 19:** A comparison between MoCA versus proposed test.

	Correct proposed test	Incorrect proposed test	Total
Correct MoCA	14	4	20
Incorrect MoCA	8	4	12
Total	22	8	30

The results demonstrate the proposed hybrid’s ability to provide high accuracy of diagnosis and early diagnosis of MCI to manage the case as early as possible and delay dementia whenever possible.

## Discussion

The correct proposed test provides higher accuracy in detecting MCI than the correct MMSE or MOCA. As the proposed test includes more objective domains than the other two tests, it can measure cortical areas’ domains, e.g., speech, apraxia, abstraction, and delayed memory testing, which also detect subcortical domains like visuospatial and executive function. Moreover, the examiner can intentionally detect the affected brain area once the patient is tested.

## Conclusion

Early diagnosis of such mental progression is essential. Several mental cognitive tests are used to check the progression of a patient’s cognitive functioning. The MoCA test checks language, visual, spatial thinking, orientation, reasoning, and Memory. Such a scoring system could quickly help determine when someone is at risk of developing Parkinson’s and Alzheimer’s. In this study, a reliable context-aware health monitoring framework based on fog computing to assess mental impairment in the elderly is introduced. This proposed framework provided a remote diagnosis of dementia diseases using fog computing technology and IoT devices. Fog computing receives sensing data from mobile applications or websites, generating real-time warning alerts based on practical data analysis.

In the proposed framework, the data were aggregated from 450 patients, 150 patients already clinically diagnosed with Alzheimer’s, 150 patients diagnosed with PD dementia, and another 150 patients with MCI (neither demented nor normal). All patients were examined cognitively by both MMSE and MOCA in the Arabic version. The aggregated dataset was utilized to make the following: (i) test the efficiency of both MMSE and MOCA scores, (ii) determine the main domains of cognition that are affected in each group, (iii) use ML for building model that could make an early diagnosis of PK and Alzheimer among MCI patients to manage the case as early as possible and prevent dementia, and (iv) providing a novel cognitive test that could quickly and accurately predict cortical and subcortical diseases. Results demonstrate the superiority of our proposed test over other tests like MoCA and MMSE. Such applications assist clinicians in providing accurate prognoses and personalized treatment, in addition to increasing patients’ quality of life.

## Supplementary Information

Supplementary Information.

## Data Availability

Raw data for dataset D1 are not publicly available to preserve individuals’ privacy and coud be accessed upon formal request . The first author could be contacted for formal request.
