# Supplementary figures and images for: Developing an explainable machine learning and fog computing-based visual rating scale for the prediction of dementia progression
Source: Sci Rep. 2025 Jul 16;15:25872. doi: 10.1038/s41598-025-06310-4 (PMC12267627; doi:10.1038/s41598-025-06310-4)

**Appendix**

**Appendix A**


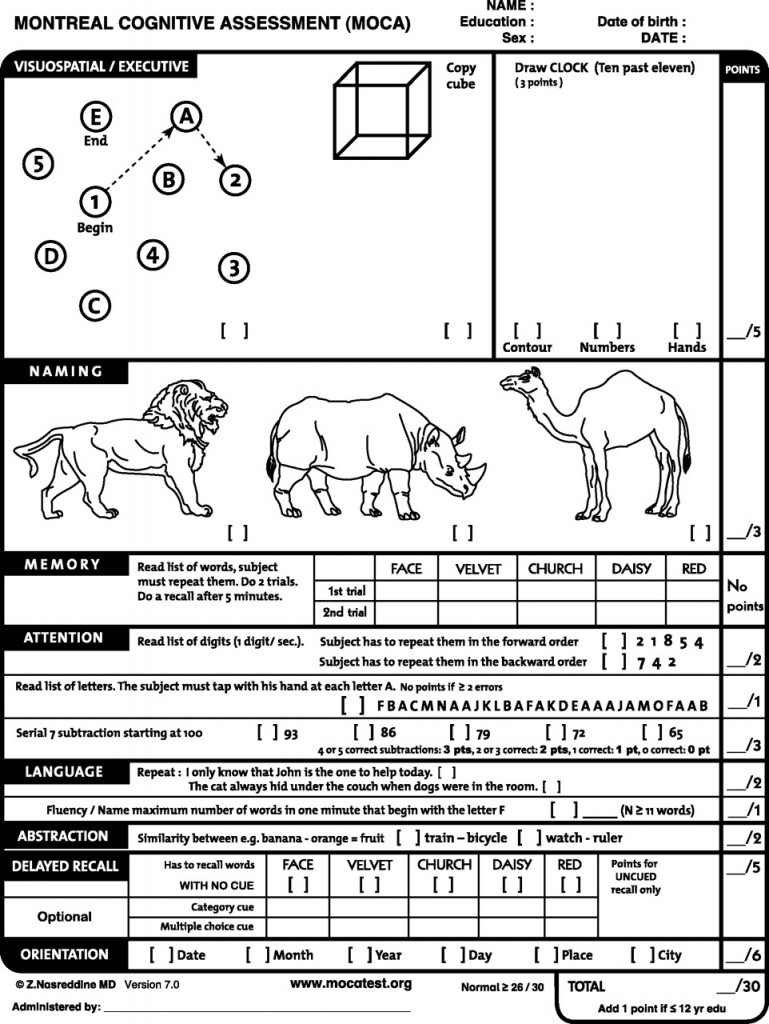

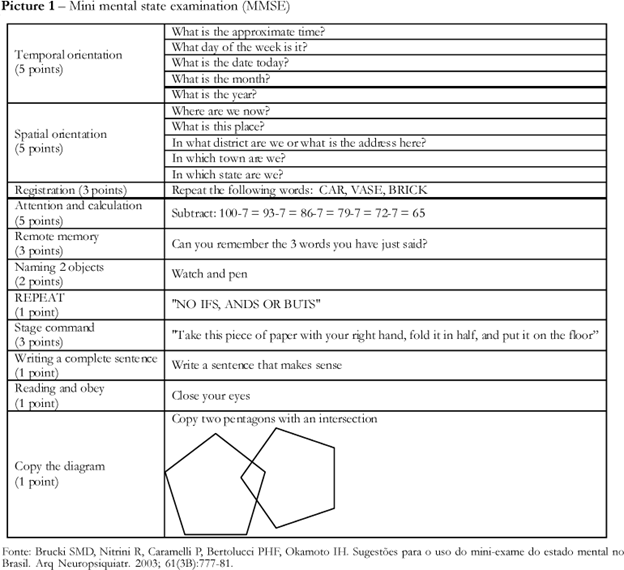

Supplement: Supplementary file 1 — Supplementary Information. [file 41598_2025_6310_MOESM1_ESM.docx]
